# *WWOX* Polymorphisms as Predictors of the Biochemical Recurrence of Localized Prostate Cancer after Radical Prostatectomy

**DOI:** 10.7150/ijms.84364

**Published:** 2023-05-21

**Authors:** Chia-Yen Lin, Chun-Li Wang, Shian-Shiang Wang, Cheng-Kuang Yang, Jian-Ri Li, Chuan-Shu Chen, Sheng-Chun Hung, Kun-Yuan Chiu, Chen-Li Cheng, Yen-Chuan Ou, Shun-Fa Yang

**Affiliations:** 1 Division of Urology, Department of Surgery, Taichung Veterans General Hospital, Taichung, Taiwan.; 2 School of Medicine, Chung Shan Medical University, Taichung, Taiwan.; 3 School of Medicine, National Yang Ming Chiao Tung University, Taipei, Taiwan.; 4 Institute of Medicine, Chung Shan Medical University, Taichung, Taiwan.; 5 Department of Family Medicine, Taichung Veterans General Hospital, Taichung, Taiwan.; 6 Department of Applied Chemistry, National Chi Nan University, Nantou, Taiwan.; 7 Department of Medicine and Nursing, Hungkuang University, Taichung, Taiwan.; 8 Department of Urology, Tung's Taichung MetroHarbor Hospital, Taichung, Taiwan.; 9 Department of Medical Research, Chung Shan Medical University Hospital, Taichung, Taiwan.

**Keywords:** WWOX, prostate cancer, polymorphism, biochemical recurrence

## Abstract

The downregulation of WW domain-containing oxidoreductase (WWOX), a tumor suppressor gene, is associated with the tumorigenesis and poor prognosis of various cancers. In this study, we investigated the associations between the polymorphisms of* WWOX*, clinicopathologic features of prostate cancer (PCa), and risk of postoperative biochemical recurrence (BCR). We evaluated the effects of five single-nucleotide polymorphisms (SNPs) of *WWOX* on the clinicopathologic features of 578 patients with PCa. The risk of postoperative BCR was 2.053-fold higher in patients carrying at least one “A” allele in *WWOX* rs12918952 than in those with homozygous G/G. Furthermore, patients with at least one polymorphic “T” allele in *WWOX* rs11545028 had an elevated (1.504-fold) risk of PCa with seminal vesicle invasion. In patients with postoperative BCR, the risks of an advanced Gleason grade and clinical metastasis were 3.317- and 5.259-fold higher in patients carrying at least one “G” allele in *WWOX* rs3764340 than in other patients. Our findings indicate the *WWOX* SNPs are significantly associated with highly aggressive pathologic features of PCa and an elevated risk of post-RP biochemical recurrence.

## Introduction

Prostate cancer (PCa) is a common and fatal malignancy. In 2022, the estimated incidence and mortality rate of PCa were 268490 and 34500 in the United States, respectively 1. Although radical prostatectomy (RP) is the treatment of choice for localized PCa, the rate of postoperative recurrence within 10 years is high at 20% to 40% 2. Prostate-specific antigen (PSA) is used as a biomarker for not only the screening and detection of PCa but also the diagnosis of postoperative tumor recurrence. After RP, the serum level of PSA is expected to decrease to an undetectable level because of the complete removal of the prostatic tissue. The PSA threshold that is indicative of biochemical recurrence (BCR) has been a subject of debate 3, 4. The American Urological Association and the European Association of Urology recommend a postoperative PSA level of ≥0.2 ng/mL in addition to a second confirmatory test for the diagnosis of BCR. However, the delineation of post-RP PSA values may not accurately reflect the nature of individual diseases. To overcome these limitations, some studies suggest for the inclusion of genetic adjustments of serum PSA level in risk assessments 5.

The WW-domain-containing oxidoreductase (WWOX) gene is identified on human chromosome 16q23.3-24.1, which contains the Common Chromosomal Fragile Site FRA16D. *WWOX* frequently undergoes chromosomal breaks and is frequently rearranged in various cancers 6-8. The WWOX protein is involved in several cellular processes, such as metabolism, DNA damage response, and tumor suppression 9-12. WWOX may interact with p53 and p73, accelerating apoptosis 13-15. The abnormal transcription and low expression of WWOX have been reported in various cancers, including breast cancer, ovarian cancer, gastric cancer, oral cancer, and PCa 16, 17. Several *in vivo* and *in vitro* studies have indicated that WWOX suppresses PCa tumorigenesis and progression by arresting the cell cycle in the G1 phase 17-20.

A single-nucleotide polymorphism (SNP) is a variation of at least 1% in a single nucleotide of the shared sequence of a gene within a population. Several genetic polymorphisms have been associated with PSA level, tumor susceptibility, tumor grade, and mortality in PCa 21-27. Moreover, WWOX SNPs have been reported in numerous cancers 28-32. However, the association between the polymorphism of *WWOX* and the risk of the postoperative BCR of PCa remains unclear. Therefore, in this study, we investigated the aforementioned association among patients with PCa in Taiwan.

## Materials and Methods

### Ethics and Patients

This study was conducted using data from a prospectively developed database of PCa. The study protocol was approved by the Institutional Review Broad of Taichung Veterans General Hospital, Taiwan (approval number: CE19062A-2). Written informed consent was obtained from all participants. We included 578 patients with prostate adenocarcinoma who underwent robotic RP with bilateral pelvic lymph node dissection at Taichung Veterans General Hospital between 2012 and 2017. The following medical data were collected: PSA level at diagnosis, Gleason score at the initial biopsy, Gleason grade group, TNM stage, other pathologic features 33, and D'Amico classification 34. To investigate the genetic influence on the risk of the post-RP BCR of PCa, we divided the cohort according to PCa recurrence and analyzed the association between the polymorphisms of* WWOX* and the clinicopathologic features of PCa.

### SNP Selection and Genomic DNA Extraction

In this study, we focused on the selection of five commonly observed polymorphisms (rs11545028, rs12918952, rs3764340, rs73569323, and rs383362) within the *WWOX* gene. These specific polymorphisms were chosen based on their well-established associations with cancer development, as demonstrated in previous studies 35, 36. Among the selected polymorphisms, rs11545028, located in the 5' untranslated region (UTR) of the *WWOX* gene, was included in our analysis. Additionally, we examined rs12918952 and rs3764340, which are situated within the gene's exonic regions. These two SNPs are of particular interest as they have the potential to lead to amino acid changes, thereby potentially compromising the tumor suppression function of WWOX 35.

The genotypic frequencies of these *WWOX* SNPs were evaluated using StepOne Real-Time PCR System (Applied Biosystems, Foster City, CA, USA) and TaqMan. The results were analyzed using SDS software (version 3.0; Applied Biosystems). The details of methods, probes, and primer sequences used in the analysis of *WWOX* variants have been reported previously 30.

Blood samples were collected from the participants before surgery. The samples (whole blood) were collected in ethylenediaminetetraacetate-containing tubes and centrifuged immediately. Genomic DNA was extracted using QIAamp DNA Blood Mini Kits (Qiagen, Valencia, CA, USA) following the manufacturer's instructions.

### Statistical Analysis

The chi-square test and Student's *t* tests were used to analyze between-group differences in demographic characteristics. Multivariate logistic regression models were used to estimate the odds ratios (ORs) and adjusted ORs (AORs) and with 95% confidence intervals (CIs) for the association between genotypic frequencies and clinicopathologic features. All analyses were performed using SAS (version 9.1, 2005, for Windows; SAS Institute, Cary, NC, USA). The statistical significance was set at p < 0.05.

## Results

### Patient Demographics

**Table 1** presents the demographic characteristics of patients with postoperative BCR of PCa and those without PCa recurrence (175 and 405 patients, respectively). Patients with postoperative BCR exhibited the following clinicopathologic characteristics. They had significantly elevated PSA level at diagnosis (>10 ng/dL), a pathologic Gleason grade group (4+5); TNM stages T3+4, N1, and M1; and poor prognostic features, namely seminal vesicle, perineural, and lymphovascular invasion. The rates of postoperative BCR per the D'Amico classification were 67.4%, 29.7%, and 2.9% in high-, intermediate-, and low-risk patients, respectively.

### Association of *WWOX* Polymorphism with Postoperative BCR

**Table 2** presents the distribution frequencies of the 5 *WWOX* genotypes (rs11545028, rs12918952, rs3764340, rs73569323, and rs383362) in 578 patients. No patient carried the homologous A/A genotype in rs12918952. However, the proportion of patients carrying heterozygous G/A in* WWOX* rs12918952 was significantly higher among patients with postoperative BCR than among those without PCa recurrence (AOR: 2.053; 95% CI: 1.088 to 3.872; p= 0.025). No prominent trend was noted in the polymorphism frequency of rs11545028, rs3764340, rs73569323, or rs383362. AORs with 95% CIs were estimated using logistical regression models after adjusting for the effects of all possible confounders (**Table 2**).

### Association of *WWOX* Polymorphism and PCa Clinical Status with Postoperative BCR

We further investigated the association between *WWOX* polymorphism and PCa clinicopathologic features in our patients (**Table 3**), even in those with postoperative BCR (**Table 4**). Patients with at least one polymorphic “T” allele in rs11545028 had a significantly higher risk of PCa with seminal vesicle invasion than did the other patients (AOR: 1.504; 95% CI: 1.012 to 2.236; p = 0.043; **Table 3**). Among patients with postoperative BCR, those carrying at least one “G” allele in rs3764340 had increased risks of an advanced Gleason grade (AOR: 3.317; 95% CI: 1.479 to 7.438; p= 0.003) and clinical metastasis (AOR: 5.259, 95% CI: 1.008 to 27.450; p = 0.030) (**Table 4**). There were no other significant associations between the remaining SNPs and clinicopathologic features of PCa in our patient cohort (Supplementary Tables 1-4).

## Discussion

Several studies have consistently revealed prominent associations between the polymorphisms of *WWOX* and the aggressive, poor prognosis of various malignancies, such as urothelial, esophageal squamous cell, hepatocellular, lung, pancreatic, and thyroid cancers 30, 31, 36-41. To the best of our knowledge, the present study is the first to investigate the association between the polymorphisms of *WWOX* and the clinicopathologic features of resectable PCa in Taiwanese men. We found a significant association between the *WWOX* SNP rs12918952 and the risk of postoperative BCR. The *WWOX* SNP rs11545028 was associated with an elevated risk of seminal invasive disease. Furthermore, *WWOX* rs3764340 was associated with increased PCa aggressiveness (high Gleason score and clinical metastasis) in patients with postoperative BCR.

Chromosomal breaks and rearrangements frequently occur in the *WWOX* of patients with cancer 6-8. Abnormal transcription and low expression of *WWOX* have been reported in several cancers with an aggressive, poor prognosis. As mentioned, WWOX is involved in PCa tumorigenesis and tumor 17-20. The SNP rs12918952 is located in the exon 5 of *WWOX*. In hepatocellular carcinoma, the “G”-to-“A” substitution of rs12918952 may accelerate vascular invasion by altering catalytic activity and downregulating the expression of WWOX mRNA 30. In our study, the risk of postoperative BCR was 2.053-fold higher in patients carrying at least one “A” allele in rs12918952 than in those with homozygous G/G. The SNP rs11545028, which is located in the exon 1 of *WWOX*, was found to be associated with a significant increase in the risk of seminal vesicle invasion. Furthermore, the SNP rs3764340, which is located in the exon 7 of *WWOX*, was found to be associated with a significant increase in the Gleason score. These SNPs in addition to preoperative PSA levels are key predictors of postoperative BCR, as indicated by several nomogram studies 42-44.

The primary objective in the treatment of surgically amenable PCa is to ensure long-term disease-free survival. Since the introduction of the trifecta concept (BCR-free survival and the recovery of continence and potency) in 2003, it has become the most popular tool in the evaluation of RP outcomes 45. However, the risk of postoperative BCR within 10 years is approximately 20% to 40% and may be up to >60% in high-risk patients with PCa 46. The rates of postoperative BCR in our cohort (67.4%, 29.7%, 2.9% in high-, intermediate-, low-risk patients, respectively) are consistent with those reported by other studies. Adjuvant and deferred radiotherapies are the treatments of choice (salvage therapy) for preventing tumor progression in patients with postoperative BCR. Many studies have indicated an association between postoperative radiotherapy and poor treatment outcomes (reduced sexual, urinary, and bowel functions) 47-49. These adverse effects of salvage therapy considerably reduce the quality of life of patients with postoperative BCR. Therefore, high accuracy must be maintained in the prediction of postoperative BCR risk and the selection of surgical intervention for these patients. Genetic adjustment has emerged as a key tool to avoid postoperative complications and maintain the normal quality of life; this tool improves the predication accuracy of postoperative BCR risk 42, 50-52.

In 2005, Andrew et al. developed and validated a robust predictive model to predict BCR after operation based on clinical and pathological features 44. However, the model did not include genetic factors. Given the highly variable behavior and clinical course of prostate cancer (PCa), it is important to adopt a personalized approach to oncologic risk stratification. Novel genetic approaches offer additional information to improve clinical decision making 53. Several studies have identified different gene signatures associated with postoperative BCR. For example, a case-control study found that a 10-gene molecular signature (HDDA10) demonstrated superior performance in predicting BCR in PCa patients after RP (AUC = 0.65) 54. Another study developed an original gene signature model that predicted 3-year BCR-free survival in PCa patients after RP (AUC = 0.836) 55. CDO1 promoter methylation has been proposed as a feasible predictive biomarker for BCR-free survival in PCa patients following RP 56. Additionally, a genetic risk score has been developed that can predict BCR by time-dependent receiver operating characteristic (t-ROC) curves in the training set (3-year AUC = 0.820, 5-year AUC = 0.809) 57. All these studies approved the significance of genetic factors in the risk of postoperative BCR. The WWOX genetic polymorphism should also be considered in genetic signature analysis, as it has been shown to be associated with prostate cancer recurrence.

Our study has some limitations. First, we only included patients with surgically amenable PCa; therefore, the findings may not be generalizable to patients with advanced PCa. Second, we lacked tumor specimens and information on the expression levels of *WWOX* in patients with PCa. Therefore, further translational studies on WWOX expression and its association with prostate cancer behavior are needed to validate the effect of genetic signature on the prognosis of tumor recurrence. Furthermore, studies on the mechanisms of *WWOX* variation and their involvement in tumor progression and recurrence are warranted.

In conclusion, our findings indicate the polymorphisms of *WWOX* are associated with highly aggressive clinicopathologic features of PCa and an elevated risk of post-RP BCR. This study may serve as a reference for future studies aimed at preventing the postoperative BCR of PCa.

## Supplementary Material

Supplementary tables.

## Figures and Tables

**Table 1:** The distributions of demographical characteristics in 578 patients with prostate cancer.

Variable	Biochemical recurrence (BCR)		
	No (n=403)	Yes (n=175)	p value
**Age at diagnosis (years**			
≤ 65	168 (41.7 %)	77 (44.0 %)	p=0.605
> 65	235 (58.3 %)	98 (56.0 %)	
**PSA at diagnosis (ng/mL)**			
≤ 10	218 (54.1 %)	52 (29.7 %)	p<0.001*
> 10	185 (45.9 %)	123 (70.3 %)	
**Pathologic Gleason grade group**			
1+2+3	366 (90.8 %)	117 (66.9 %)	p<0.001*
4+5	37 (9.2 %)	58 (33.1 %)	
**Clinical T stage**			
1+2	368 (91.3 %)	132 (75.4 %)	p<0.001*
3+4	35 (8.7 %)	43 (24.6 %)	
**Clinical N stage**			
N0	396 (98.3 %)	169 (96.6 %)	p=0.208
N1	7 (1.7 %)	6 (3.4 %)	
**Clinical M stage**			
M0	399 (99.0 %)	169 (96.6 %)	p=0.039*
M1	4 (1.0 %)	6 (3.4 %)	
**Pathologic T stage**			
2	266 (66.0 %)	40 (22.9 %)	p<0.001*
3+4	137 (34.0 %)	135 (77.1 %)	
**Pathologic N stage**			
N0	392 (97.3 %)	137 (78.3 %)	p<0.001*
N1	11 (2.7 %)	38 (21.7 %)	
**Seminal vesicle invasion**			
No	363 (90.1 %)	88 (50.3 %)	p<0.001*
Yes	40 (9.9 %)	87 (49.7 %)	
**Perineural invasion**			
No	140 (34.7 %)	15 (8.6%)	p<0.001*
Yes	263 (65.3 %)	160 (91.4 %)	
**Lymphovascular invasion**			
No	372 (92.3 %)	109 (62.3 %)	p<0.001*
Yes	31 (7.7 %)	66 (37.7 %)	
**D'Amico classification**			
Low risk	55 (13.6 %)	5 (2.9 %)	p<0.001*
Intermediate risk	167 (41.5 %)	52 (29.7 %)	
High risk	181 (44.9 %)	118 (67.4 %)	

**Table 2:** Distribution frequency of *WWOX* genotypes in 578 patients with prostate cancer.

Variable	Biochemical recurrence (BCR)			
	No (n=403)	Yes (n=175)	OR (95% CI)	AOR (95% CI)
**rs11545028**				
CC	239 (59.3%)	102 (58.3%)	1.00	1.00
CT	144 (35.7%)	64 (36.6%)	1.041 (0.716-1.515)	0.916 (0.586-1.432)
TT	20 (5.0%)	9 (5.1%)	1.054 (0.464-2.394)	0.671 (0.246-1.826)
CT+TT	164 (40.7%)	73 (41.7%)	1.043 (0.727-1.495)	0.884 (0.574-1.360)
**rs12918952**				
GG	365 (90.6%)	148 (84.6%)	1.00	1.00
GA	38 (9.4%)	27 (15.4%)	**1.752 (1.032-2.974)** p=0.038*	**2.053 (1.088-3.872)** p=0.026*
AA	0 (0%)	(0%)	---	---
GA+AA	38 (9.4%)	27 (15.4%)	**1.752 (1.032-2.974)** p=0.038*	**2.053 (1.088-3.872)** p=0.026*
**rs3764340**				
CC	337 (83.6%)	145 (82.9%)	1.00	1.00
CG	61 (15.1%)	27 (15.4%)	1.029 (0.628-1.684)	0.972 (0.537-1.759)
GG	5 (1.3%)	3 (1.7%)	1.394 (0.329-5.912)	3.402 (0.638-18.146)
CG+GG	66 (16.4%)	30 (17.1%)	1.056 (0.658-1.696)	1.077 (0.610-1.902)
**rs73569323**				
CC	388 (96.3%)	167 (95.4%)	1.00	1.00
CT	15 (3.7%)	8 (4.6%)	1.239 (0.515-2.979)	1.784 (0.675-4.719)
TT	0 (0%)	0 (0%)	---	---
CT+TT	15 (3.7%)	8 (4.6%)	1.239 (0.515-2.979)	1.784 (0.675-4.719)
**rs383362**				
GG	310 (76.9%)	128 (73.1%)	1.00	1.00
GT	86 (21.3%)	45 (25.7%)	1.267 (0.837-1.920)	1.012 (0.618-1.658)
TT	7 (1.8%)	2 (1.2%)	0.692 (0.142-3.376)	0.732 (0.099-5.405)
GT+TT	93 (23.1%)	47 (26.9%)	1.224 (0.815-1.838)	0.997 (0.614-1.619)

The odds ratios (ORs) and with their 95% confidence intervals (CIs) were estimated by logistic regression models. The adjusted odds ratios (AORs) with their 95% confidence intervals (CIs) were estimated by multiple logistic regression models after controlling for Age at diagnosis, PSA, pathologic Gleason grade group, clinical T stage, clinical N stage, clinical M stage, pathologic T stage, seminal vesicle invasion, perineural invasion, lymphovascular invasion and D'Amico classification. * p value < 0.05 as statistically significant.

**Table 3:** Odds ratio (OR) and 95% confidence interval (CI) of clinical status and *WWOX* rs11545028 genotypic frequencies in 578 patients with prostate cancer.

Variable	Genotypic frequencies			
rs11545028	CC (N=341)	CT+TT (N=237)	OR (95% CI)	p value
**PSA at diagnosis (ng/mL)**				
≤ 10	156 (45.7 %)	114 (48.1 %)	1.00	p=0.577
> 10	185 (54.3 %)	123 (51.9 %)	0.910 (0.653-1.268)	
**Pathologic Gleason grade group**				
1+2+3	289 (84.8%)	194 (81.9%)	1.00	p=0.356
4+5	52 (15.2%)	43 (18.1%)	1.232 (0.791-1.919)	
**Clinical T stage**				
1+2	298 (87.4%)	202 (85.2%)	1.00	p=0.455
3+4	43 (12.6%)	35 (14.8%)	1.201 (0.743-1.942)	
**Clinical N stage**				
N0	333 (97.7 %)	232 (97.9 %)	1.00	p=0.851
N1	8 (2.3 %)	5 (2.1 %)	0.897 (0.290-2.777)	
**Clinical M stage**				
M0	336 (98.5 %)	232 (97.9 %)	1.00	p=0.560
M1	5 (1.5 %)	5 (2.1 %)	1.448 (0.415-5.059)	
**Pathologic T stage**				
2	186 (54.5%)	120 (50.6%)	1.00	p=0.354
3+4	155 (45.5%)	117 (49.4%)	1.170 (0.839-1.631)	
**Pathologic N stage**				
N0	315 (92.4%)	214 (90.3%)	1.00	p=0.377
N1	26 (7.6%)	23 (9.7%)	1.302 (0.724-2.343)	
**Seminal vesicle invasion**				
No	276 (80.9%)	175 (73.8%)	1.00	p=0.043*
Yes	65 (19.1%)	62 (26.2%)	1.504 (1.012-2.236)	
**Perineural invasion**				
No	92 (27.0%)	63 (26.6%)	1.00	p=0.916
Yes	249 (73.0%)	174 (73.4%)	1.020 (0.702-1.484)	
**Lymphovascular invasion**				
No	284 (83.3%)	197 (83.1%)	1.00	p=0.959
Yes	57 (16.7%)	40 (16.9%)	1.012 (0.649-1.576)	
**D'Amico classification**				
Low/Intermediate risk	161 (47.2%)	118 (49.8%)	1.00	p=0.542
High risk	180 (52.8%)	119 (50.2%)	0.902 (0.647-1.257)	

The ORs with analyzed by their 95% CIs were estimated by logistic regression models. * p value < 0.05 as statistically significant.

**Table 4:** Odds ratio (OR) and 95% confidence interval (CI) of clinical status and *WWOX* rs3764340 genotypic frequencies in 175 prostate cancer patients with biochemical recurrence.

Variable	Genotypic frequencies			
rs3764340	CC (N=145)	CG +GG (N=30)	OR (95% CI)	p value
**PSA at diagnosis (ng/mL)**				
≤ 10	41 (28.3 %)	11 (36.7 %)	1.00	p=0.360
> 10	104 (71.7 %)	19 (63.3 %)	0.681 (0.298-1.555)	
**Pathologic Gleason grade group**				
1+2+3	104 (71.7%)	13 (43.3%)	1.00	p=0.003*
4+5	41 (28.3%)	17 (56.7%)	3.317 (1.479-7.438)	
**Clinical T stage**				
1+2	108 (74.5%)	24 (80.0%)	1.00	p=0.523
3+4	37 (25.5%)	6 (20.0%)	0.730 (0.277-1.924)	
**Clinical N stage**				
N0	139 (95.9 %)	30 (100 %)	1.00	p=0.257
N1	6 (4.1 %)	0 (0 %)	---	
**Clinical M stage**				
M0	142 (97.9 %)	27 (90.0 %)	1.00	p=0.030*
M1	3 (2.1 %)	3 (10.0 %)	5.259 (1.008-27.450)	
**Pathologic T stage**				
2	33 (22.8%)	7 (23.3%)	1.00	p=0.946
3+4	112 (77.2%)	23 (76.7%)	0.968 (0.382-2.456)	
**Pathologic N stage**				
N0	115 (79.3%)	22 (73.3%)	1.00	p=0.470
N1	30 (20.7%)	8 (26.7%)	1.394 (0.565-3.440)	
**Seminal vesicle invasion**				
No	75 (51.7%)	13 (43.3%)	1.00	p=0.403
Yes	70 (48.3%)	17 (56.7%)	1.401 (0.634-3.094)	
**Perineural invasion**				
No	11 (7.6%)	4 (13.3%)	1.00	p=0.306
Yes	134 (92.4%)	26 (86.7%)	0.534 (0.158-1.806)	
**Lymphovascular invasion**				
No	89 (61.4%)	20 (66.7%)	1.00	p=0.587
Yes	56 (38.6%)	10 (33.3%)	0.795 (0.347-1.821)	
**D'Amico classification**				
Low/Intermediate risk	49 (33.8%)	8 (26.7%)	1.00	p=0.448
High risk	96 (66.2%)	22 (73.3%)	1.404 (0.583-3.382)	

The ORs with analyzed by their 95% CIs were estimated by logistic regression models. * p value < 0.05 as statistically significant.
