# Supplementary material for: WWOX Polymorphisms as Predictors of the Biochemical Recurrence of Localized Prostate Cancer after Radical Prostatectomy
Source: Int J Med Sci. 2023 May 21;20(7):969–75. doi: 10.7150/ijms.84364 (PMC10266044; doi:10.7150/ijms.84364)
Supplement: Supplementary file 1 — Supplementary tables. [file ijmsv20p0969s1.pdf]

Supplementary Table 1. Association of clinical status and *WWOX* rs12918952 genotypic frequencies in 578 patients with prostate cancer.

**Variable**	**Genotypic frequencies**		
**rs12918952**	**GG (N=513)**	**GA+AA (N=65)**	**p value**
**PSA at diagnosis (ng/mL)**			
≤ 10	243 (47.4 %)	27 (41.5 %)	p=0.375
> 10	270 (52.6 %)	38 (58.5 %)	
**Pathologic Gleason grade group**			
1+2+3	432 (84.2%)	51 (78.5%)	p=0.239
4+5	81 (15.8%)	14 (21.5%)	
**Clinical T stage**			
1+2	444 (86.5%)	56 (86.2%)	p=0.930
3+4	69 (13.5%)	9 (13.8%)	
**Clinical N stage**			
N0	502 (97.9 %)	63 (96.9 %)	p=0.633
N1	11 (2.1 %)	2 (3.1 %)	
**Clinical M stage**			
M0	506 (98.6 %)	62 (95.4 %)	p=0.058
M1	7 (1.4 %)	3 (4.6 %)	
**Pathologic T stage**			
2	272 (53.0%)	34 (52.3%)	p=0.914
3+4	241 (47.0%)	31 (47.7%)	
**Pathologic N stage**			
N0	473 (92.2%)	56 (86.2%)	p=0.099
N1	40 (7.8%)	9 (13.8%)	
**Seminal vesicle invasion**			
No	400 (78.0%)	51 (78.5%)	p=0.929
Yes	113 (22.0%)	14 (21.5%)	
**Perineural invasion**			
No	135 (26.3%)	20 (30.8%)	p=0.445
Yes	378 (73.7%)	45 (69.2%)	
**Lymphovascular invasion**			
No	429 (83.6%)	52 (80.0%)	p=0.461
Yes	84 (16.4%)	13 (20.0%)	
**D'Amico classification**			
Low/Intermediate risk	246 (48.0%)	33 (50.8%)	p=0.669
High risk	267 (52.0%)	32 (49.2%)	

Supplementary Table 2. Association of clinical status and *WWOX* rs3764340 genotypic frequencies in 578 patients with prostate cancer.

**Variable**	**Genotypic frequencies**		
**rs3764340**	**CC (N=482)**	**CG+GG (N=96)**	**p value**
**PSA at diagnosis (ng/mL)**			
≤ 10	227 (47.1 %)	43 (44.8 %)	p=0.679
> 10	255 (52.9 %)	53 (55.2 %)	
**Pathologic Gleason grade group**			
1+2+3	408 (84.6%)	75 (78.1%)	p=0.115
4+5	74 (15.4%)	21 (21.9%)	
**Clinical T stage**			
1+2	415 (86.1%)	85 (88.5%)	p=0.522
3+4	67 (13.9%)	11 (11.5%)	
**Clinical N stage**			
N0	470 (97.5 %)	95 (99.0 %)	p=0.382
N1	12 (2.5 %)	1 (1.0 %)	
**Clinical M stage**			
M0	475 (98.5 %)	93 (96.9 %)	p=0.251
M1	7 (1.5 %)	3 (3.1 %)	
**Pathologic T stage**			
2	256 (53.1%)	50 (52.1%)	p=0.854
3+4	226 (46.9%)	46 (47.4%)	
**Pathologic N stage**			
N0	443 (91.9%)	86 (89.6%)	p=0.455
N1	39 (8.1%)	10 (10.4%)	
**Seminal vesicle invasion**			
No	377 (78.2%)	74 (77.1%)	p=0.807
Yes	105 (21.8%)	22 (22.9%)	
**Perineural invasion**			
No	125 (25.9%)	30 (31.2%)	p=0.283
Yes	357 (74.1%)	66 (68.8%)	
**Lymphovascular invasion**			
No	400 (83.0%)	81 (84.4%)	p=0.740
Yes	82 (17.0%)	15 (15.6%)	
**D'Amico classification**			
Low/Intermediate risk	237 (49.2%)	42 (43.8%)	p=0.332
High risk	245 (50.8%)	54 (56.3%)	

Supplementary Table 3. Association of clinical status and *WWOX* rs73569323 genotypic frequencies in 578 patients with prostate cancer.

Variable	Genotypic frequencies		
**rs73569323**	**CC (N=555)**	**CT+TT (N=23)**	**p value**
**PSA at diagnosis (ng/mL)**			
≤ 10	257 (46.3 %)	13 (56.5 %)	p=0.336
> 10	298 (53.7 %)	10 (43.5 %)	
**Pathologic Gleason grade group**			
1+2+3	461 (83.1%)	22 (95.7%)	p=0.110
4+5	94 (16.9%)	1 (4.3%)	
**Clinical T stage**			
1+2	478 (86.1%)	22 (95.7%)	p=0.190
3+4	77 (13.9%)	1 (4.3%)	
**Clinical N stage**			
N0	542 (97.7 %)	23 (100 %)	p=0.458
N1	13 (2.3 %)	0 (0.0 %)	
**Clinical M stage**			
M0	545 (98.2 %)	23 (100 %)	p=0.516
M1	10 (1.8 %)	0 (0.0 %)	
**Pathologic T stage**			
2	294 (53.0%)	12 (52.2%)	p=0.940
3+4	261 (47.0%)	11 (47.8%)	
**Pathologic N stage**			
N0	507 (91.4%)	22 (95.7%)	p=0.468
N1	48 (8.6%)	1 (4.3%)	
**Seminal vesicle invasion**			
No	431 (77.7%)	20 (87.0%)	p=0.291
Yes	124 (22.3%)	3 (13.0%)	
**Perineural invasion**			
No	150 (27.0%)	5 (21.7%)	p=0.575
Yes	405 (73.0%)	18 (78.3%)	
**Lymphovascular invasion**			
No	462 (83.2%)	19 (82.6%)	p=0.936
Yes	93 (16.8%)	4 (17.4%)	
**D'Amico classification**			
Low/Intermediate risk	265 (47.7%)	14 (60.9%)	p=0.217
High risk	290 (52.3%)	9 (39.1%)	

Supplementary Table 4. Association of clinical status and *WWOX* rs383362 genotypic frequencies in 578 patients with prostate cancer.

Variable	Genotypic frequencies		
rs383362	GG (N=438)	GT+TT (N=140)	p value
**PSA at diagnosis (ng/mL)**			
≤ 10	208 (47.5 %)	62 (44.3 %)	p=0.508
> 10	230 (52.5 %)	78 (55.7 %)	
**Pathologic Gleason grade group**			
1+2+3	367 (83.8%)	116 (82.9%)	p=0.795
4+5	71 (16.2%)	24 (17.1%)	
**Clinical T stage**			
1+2	379 (86.5%)	121 (86.4%)	p=0.976
3+4	59 (13.5%)	19 (13.6%)	
**Clinical N stage**			
N0	428 (97.7 %)	137 (97.9 %)	p=0.922
N1	10 (2.3 %)	3 (2.1 %)	
**Clinical M stage**			
M0	428 (97.7 %)	140 (100 %)	p=0.071
M1	10 (2.3 %)	0 (0.0 %)	
**Pathologic T stage**			
2	241 (55.2%)	65 (46.4%)	p=0.062
3+4	197 (44.8%)	75 (53.6%)	
**Pathologic N stage**			
N0	400 (91.3%)	129 (92.1%)	p=0.762
N1	38 (8.7%)	11 (7.9%)	
**Seminal vesicle invasion**			
No	349 (79.7%)	102 (72.9%)	p=0.090
Yes	89 (20.3%)	38 (27.1%)	
**Perineural invasion**			
No	123 (28.1%)	32 (22.9%)	p=0.224
Yes	315 (71.9%)	108 (77.1%)	
**Lymphovascular invasion**			
No	370 (84.5%)	111 (79.3%)	p=0.153
Yes	68 (15.5%)	29 (20.7%)	
**D’Amico classification**			
Low/Intermediate risk	218 (49.8%)	61 (43.6%)	p=0.201
High risk	220 (50.2%)	79 (56.4%)	
